# Methicillin- and Vancomycin-Resistant *Staphylococcus aureus* From Humans and Ready-To-Eat Meat: Characterization of Antimicrobial Resistance and Biofilm Formation Ability

**DOI:** 10.3389/fmicb.2021.735494

**Published:** 2022-02-08

**Authors:** Taisir Saber, Mohamed Samir, Rasha M. El-Mekkawy, Eman Ariny, Sara Ramadan El-Sayed, Gamal Enan, Sawasn H. Abdelatif, Ahmed Askora, Abdallah M. A. Merwad, Yasmine H. Tartor

**Affiliations:** ^1^Department of Medical Microbiology and Immunology, Faculty of Medicine, Zagazig University, Zagazig, Egypt; ^2^Department of Clinical Laboratory Sciences, College of Applied Medical Sciences, Taif University, Taif, Saudi Arabia; ^3^Department of Zoonoses, Faculty of Veterinary Medicine, Zagazig University, Zagazig, Egypt; ^4^Department of Botany and Microbiology, Faculty of Science, Zagazig University, Zagazig, Egypt; ^5^Department of Pediatrics, Faculty of Medicine, Zagazig University, Zagazig, Egypt; ^6^Department of Microbiology, Faculty of Veterinary Medicine, Zagazig University, Zagazig, Egypt

**Keywords:** VRSA, MRSA, multidrug resistance, biofilm, ready-to-eat meat, *S. aureus*, food handlers, patients

## Abstract

Methicillin-resistant and vancomycin-resistant *Staphylococcus aureus* (MRSA and VRSA) are zoonotic life-threatening pathogens, and their presence in food raises a public health concern. Yet, scarce data are available regarding MRSA and VRSA in both ready-to-eat (RTE) meat and food handlers. This study was undertaken to determine the frequency, antimicrobial resistance, and biofilm-forming ability of MRSA and VRSA isolated from RTE meat (shawarma and burger) and humans (food handlers, and hospitalized patients) in Zagazig city, Sharkia Governorate, Egypt. We analyzed 176 samples (112 human samples: 72 from hospitalized patients and 40 from food handlers, 64 RTE meat samples: 38 from shawarma and 26 from burger). Using phenotypic, PCR-based identification of *nuc* gene and matrix-assisted laser desorption ionization-time of flight mass spectrometry (MALDI-TOF MS), 60 coagulase-positive *S. aureus* (COPS) isolates were identified in the samples as follow: RTE meat (15/64, 23.4%), hospitalized patients (33/72, 45.8%) and food handlers (12/40, 30%). All the COPS isolates were *mecA* positive (and thus were classified as MRSA) and multidrug resistant with multiple antibiotic resistance indices ranging from 0.25 to 0.92. Overall, resistance to cefepime (96.7%), penicillin (88.3%), were common, followed by ampicillin-sulbactam (65%), ciprofloxacin (55%), nitrofurontoin (51.7%), and gentamicin (43.3%). VRSA was detected in 30.3% of COPS hospitalized patient’s isolates, 26.7% of COPS RTE meat isolates and 25% of COPS food handler’s isolates. *VanA*, *vanB*, or both genes were detected in 64.7, 5.9, and 29.4% of all VAN-resistant isolates, respectively. The majority of the COPS isolates (50/60, 83.3%) have biofilm formation ability and harbored *icaA* (76%), *icaD* (74%), *icaC* (50%), and *icaB* (46%) biofilm-forming genes. The *bap* gene was not detected in any of the isolates. The ability of MRSA and VRSA isolates to produce biofilms in addition to being resistant to antimicrobials highlight the danger posed by these potentially virulent microorganisms persisting in RTE meat, food handlers, and patients. Taken together, good hygiene practices and antimicrobial surveillance plans should be strictly implemented along the food chain to reduce the risk of colonization and dissemination of MRSA and VRSA biofilm-producing strains.

## Introduction

*Staphylococcus aureus* is an opportunistic human and animal pathogen causing food intoxication and a variety of infections ranging from skin and soft tissue infections to serious diseases including endocarditis, septicemia, osteomyelitis, pneumonia and toxic shock syndrome (Chen and Huang, 2014; Otto, 2014). The drug resistance of *S. aureus* has gradually increased in the recent decades due to the misuse of antibiotics (e.g., using antibiotics without prescription, uncontrolled doses, and useless application of drugs), which resulted in bacterial evolution (Gharieb et al., 2020; Guo et al., 2020). Consequently, multidrug-resistant *S. aureus* especially methicillin-resistant (MRSA) strains are a major human health concern causing severe morbidity and mortality, in particular in hospitals (hospital-associated MRSA; HA-MRSA), and in healthy persons (community-associated MRSA; CA-MRSA) (Weber, 2005). MRSA, one of twelve priority pathogens that threaten human health as classified by the World Health Organization (WHO), has been increasingly detected in food products (Craft et al., 2019; Li et al., 2019). Animal- derived foods serve as vehicles for the transmission of livestock- associated MRSA (LA-MRSA) (Mossong et al., 2015; Anjum et al., 2019). Further, human-mediated contamination of carcasses, meat product or ready-to-eat (RTE) foods at abattoirs, meat processing plants or during handling may be a source of MRSA and could represent potential risk for consumers (Hadjirin et al., 2015; Li et al., 2019).

Apart from the studies focused on LA-MRSA, the prevalence of staphylococci in RTE foods is receiving widespread attention due to the increasing number of food poisoning cases (Chajecka-Wierzchowska et al., 2015; Yang et al., 2017). MRSA strains exhibit resistance to various antimicrobials such as penicillins, cephalosporins, and carbapenem through the acquisition of the mobile staphylococcal cassette chromosome mec (SCCmec) that carries *mecA* gene, which encodes for an altered penicillin binding proteins (PBP2a or PBP2′), thereby making their treatment difficult (Hiramatsu et al., 2014). Vancomycin has long been considered the last-resort treatment for MRSA infections (Holmes et al., 2015). However, its excessive use resulted in the emergence of vancomycin-intermediate *S. aureus* (VISA), vancomycin-resistant *S. aureus* (VRSA), and heterogeneous vancomycin-intermediate *S. aureus* (hVISA) strains (Amberpet et al., 2019). In addition to antimicrobial resistance, MRSA strains are able to form biofilm as a fitness and survival mechanism that is mediated by the strong adhesion, increase in drug resistance and reduction in the effectiveness of sanitizers (Craft et al., 2019). In the presence of biofilm, resistance of *S. aureus* to antimicrobials was reported to increase to 1,000 times that of planktonic cells (Guo et al., 2020). Therefore, MRSA biofilm formation is an important persistence and dissemination mechanism of food contamination (Vergara et al., 2017; Avila-Novoa et al., 2021). Biofilm development is thought to be a two-step process in which bacteria adhere to a surface via a capsular antigen, capsular polysaccharide/adhesin (PS/A), and then multiply to form a multilayered biofilm, which is triggered by the synthesis of polysaccharide intercellular adhesin (PIA) from β-1, 6- linked N-acetyld-glucosamines (Periasamy et al., 2012). The intercellular adhesion (*ica*) locus is comprised of *icaA*, *icaD*, *icaB*, and *icaC* genes that mediate the production of PIA and PS/A (McKenney et al., 1998). The *icaA* gene encodes N-acetylglucosaminyltransferase enzyme. Furthermore, *icaD* has been shown to play a key role in the maximum expression of this enzyme, which leads to capsular polysaccharide phenotypic expression (Arciola et al., 2001). The *icaC* functions as a polysaccharide receptor (McKenney et al., 1998). Furthermore, the biofilm-associated protein (*Bap*) is required for *S. aureus* initial attachment and biofilm development (Cucarella et al., 2004). Vaccine candidates consisting of several antigens related to multiple stages of biofilm formation and biofilm-related macromolecules have been proposed as a strategy for prevention and treatment of staphylococcal infection (Mirzaei et al., 2016, 2019, 2021; Bahonar et al., 2019; Gholami et al., 2019).

Indeed, data on the prevalence and biofilm formation by MRSA and VRSA in RTE food of animal origin remain scarce. In Egypt, most of the studies on the prevalence of MRSA isolates in RTE meat has focused on their antimicrobial resistance profile (Saad et al., 2019; Mahros et al., 2021), yet these studies did not analyze biofilm formation potential by the isolates, did not analyze the clustering pattern of such isolates in this important source and no studies exist that included isolates from humans. Here, we provide a characterization of a set of MRSA and VRSA isolates from humans (patients and food handlers) and RTE meat (shawarma and burger) focusing on their frequency, antimicrobial resistance phenotype, biofilm formation ability and how antimicrobial resistance and biofilm formation, as fitness traits, could be linked.

## Materials and Methods

### Study Design and Sample Collection

**FIGURE 1:**
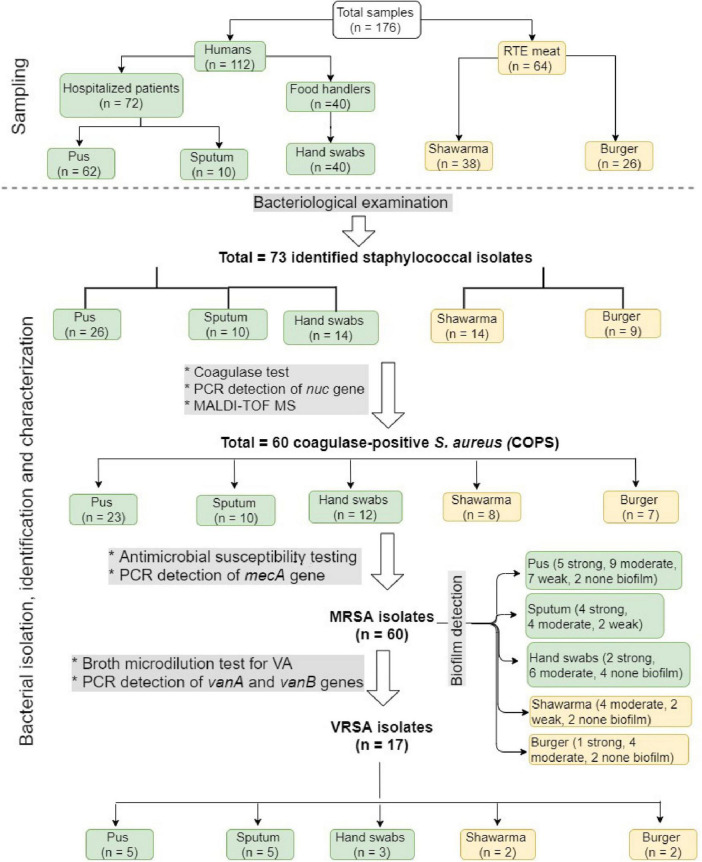
A flow chart showing the overall study design, including sampling and bacterial isolation, identification, and characterization.

Figure 1 shows the overall study design with respect to sampling, bacterial isolation, and characterization. During the period from February 2018 to January 2019, 176 samples were collected for isolation and characterization of *S. aureus*. These included 64 RTE meat samples from both shawarma (*n* = 38) and burger (*n* = 26) sandwiches that were collected from six different restaurants in Zagazig city, Sharkia Governorate, Egypt. Moreover, 112 human samples were collected; including 72 samples from patients hospitalized at Zagazig University hospital (62 pus samples from abscesses and wounds and 10 sputum samples) and 40 hand swab samples from food handlers, who, at the time of examination, were clinically free from any bacterial skin infections. All samples were collected aseptically and immediately transferred in an icebox to the laboratory for isolation of *Staphylococcus* species. All participants were informed about the nature of the study and signed informed consent approving the use of their specimens for research purposes. The protocol of this study has been reviewed and approved by ZU-IACUC Committee (approval number ZU-IACUC/2/F/11/2019).

### Isolation and Identification of *S. aureus*

The RTE meat and human samples were subjected to the standard microbiological techniques for isolation and identification of *S. aureus* (Cheesbrough, 2006; Aureus, 2016). Following National Food Safety Standards of China document GB 4789.10-2016 (Aureus, 2016), 25 g of each RTE meat sample was homogenized in 225 mL of tryptic soy broth (TSB, Thermo Fisher Scientific Oxoid Ltd., Basingstoke, Hampshire, United Kingdom) with 10% sodium chloride. The homogenate (1 mL) was added to 9 mL of TSB in a sterile tube then incubated at 37°C for 24 h. One hundred μL of food homogenate, hand swabs, swabs from pus as well as sputum samples were plated on Baird-Parker agar (BPA) medium supplemented with an egg yolk–tellurite emulsion (Thermo Fisher Scientific Oxoid Ltd., Basingstoke, Hampshire, United Kingdom) with and without acriflavine supplementation (7 μg/mL; Acros Organics, Morris Plains, NJ, United States) and incubated at 37°C for 48 h. The presumptive black-colored colonies surrounded by a clear halo zone were subcultured onto mannitol salt agar medium (Thermo Fisher Scientific Oxoid Ltd., Basingstoke, Hampshire, United Kingdom), blood agar, and subjected to Gram staining, catalase, coagulase, and oxidation/fermentation tests (Roberson et al., 1992). Also, the recovered isolates were identified using the MALDI TOF MS (BioMeriéux, Marcy I’Etoile, France) and MALDI-TOF MS running MYLA 3.1.0-15 software (BioMérieux, Inc., Marcy I’Etoile, France) according to Luo et al. (2015) and were further confirmed by *nuc* gene amplification as described previously (Brakstad et al., 1992) (Supplementary Table 1). The identified isolates were preserved at −20°C in brain heart infusion broth (BHI, Oxoid, Ltd., Basingstoke, Hampshire, United Kingdom) with 30% glycerol for further analysis.

### Antimicrobial Susceptibility Testing

Kirby-Bauer disk diffusion test recommended by the Clinical and laboratory standards institute (CLSI) (CLSI, 2018) was employed for phenotypically testing the susceptibility of *S. aureus* isolates to 12 antimicrobial agents commonly used in clinical and veterinary medicine including: penicillin (P, 10 μg, Oxoid, Basingstoke, United Kingdom), methicillin (Me, 5 μg), nitrofurantoin (F, 300 μg), ampicillin/sulbactam (SAM, 20 μg), cefuroxime (CXM, 30 μg), ciprofloxacin (CIP, 5 μg), cefepime (FEP, 50 μg), trimethoprim/sulfamethoxazole (SXT, 23.75/1.25 μg) gentamycin (CN, 10 μg), clindamycin (DA, 2 μg), erythromycin (E, 15 μg), and vancomycin (VA, 30 μg). The results were interpreted according to CLSI guidelines in the reference mentioned before. *S. aureus* ATCC 25923 reference strain was included in the test as a control. The multiple antibiotic resistance (MAR) index was estimated by dividing the number of antimicrobial agents to which the isolate displayed resistance by the total number of tested antimicrobials (Krumperman, 1983). MAR index value > 0.2 identifies the isolate that originates from high-risk source of contamination where antibiotics are extensively used. The isolates were also examined using broth microdilution test for determination of vancomycin (Sigma-Aldrich, United States) minimum inhibitory concentrations (MICs) according to the CLSI guidelines (CLSI, 2018). Isolates, which have MIC ≤ 2 μg/mL was considered as vancomycin-susceptible *S. aureus* (VSSA), VISA: 4–8 μg/mL, and VRSA: MIC >8 μg/mL.

### PCR Detection of Methicillin- and Vancomycin- Resistance Genes

Genomic DNA was extracted from 24 h cultures of phenotypic MRSA and VRSA isolates in BHI broth using the QIAamp DNA Mini kit (Qiagen, GmbH, Germany) according to the manufacturer’s instructions. The DNA quantity and purity were assessed using NanoDrop™ 1000 spectrophotometer (Thermo Fisher Scientific, Waltham, MA, United States). Methicillin resistance gene (*mecA*) and vancomycin resistance genes (*vanA* and *vanB*) were detected by PCR using oligonucleotide primers (Supplementary Table 1) as previously described (Patel et al., 1997; Kariyama et al., 2000; McClure et al., 2006). PCR amplification was performed in a T3 Thermal cycler (Biometra GmbH, Göttingen, Germany) using 6 μL of the extracted DNA (equalized at 100 ng/μL), 12.5 μL of 2X EmeraldAmp GT PCR master mix (Takara, Japan), and 1 μL (20 pmol) of both forward and reverse primers (Metabion, Germany), and nuclease-free water was added up to 25 μL. *S. aureus* ATCC 33591 (MRSA) and ATCC 29213 (methicillin-susceptible) strains were used as positive and negative controls, respectively. *Enterococcus faecium* ATCC 51559 and *E. faecalis* ATCC 51299 were used as a *vanA*- and *vanB*-positive control strains, respectively. The PCR-amplified products were analyzed by electrophoresis on a 1.5% agarose gel containing 0.5 μg/mL ethidium bromide and visualized using AlphaDigiDoc^®^ RT gel documentation system (Alpha Innotech Corp., San Leandro, CA, United States). A 100 bp ladder (Cat. No. SM0243, Fermentas, United States) was used as a molecular size marker.

### Detection of Biofilm Formation Ability and Biofilm Genes

Biofilm formation ability of coagulase-positive *S. aureus* (COPS) isolates (*n* = 60) was assessed and analyzed using 96 wells flat-bottom polystyrene microtiter plates (Techno Plastic Products, Switzerland) as previously described (Stepanovic et al., 2007). The fresh culture of each isolate in TSB (200 μL, 10^6^ CFU/mL) was inoculated into wells of a sterile microtiter plate and incubated at 37°C for 24 h. TSB without bacteria and *S. aureus* ATCC 25923 were used as negative and positive controls, respectively. The content of wells was aspirated, and each well was washed three times with 200 μL of phosphate buffer saline (PBS, pH 7.3) to remove non-adherent cells. The plates were drained and air-dried for 15 min. The biofilms were stained with 150 μL of 0.1% crystal violet (Fluka AG, Buchs, Switzerland) for 30 min, and thereafter washed twice with PBS and the plate was air-dried. The stain bound to the cells was resolubilized with 150 μL of 95% ethanol for 45 min and the optical density (OD) was determined at a wavelength of 570 nm by ELISA reader (Awareness Technologies stat fax 2100, CA, United States). The isolates were tested in triplicate and the test was performed three times. Therefore, the average OD values ± standard deviations (SD) were calculated for the tested isolates and negative controls. For interpretation of biofilm production, cut-off value of the OD (ODc) was calculated: ODc = average OD of negative control + (3 × SD of negative control) and accordingly the isolates were classified into the following categories: non-producer OD ≤ ODc, weak (ODc < OD ≤ 2 × ODc), moderate (2 × ODc < OD ≤ 4 × ODc), and strong biofilm producers (4 × ODc < OD). Biofilm-producing isolates were further investigated for biofilm-related genes listed in supplementary Table 1 (*icaA*, *icaB, icaD*, *icaC*, and *bap*) as previously described (Vasudevan et al., 2003; Cucarella et al., 2004; Kiem et al., 2004).

**TABLE 1:** Antibiograms, biofilm formation ability, vancomycin MIC, and resistance genes of the 60 MRSA isolates.

Antibiotype No.	No. of isolates/pattern	Source	No. of resistant antimicrobials	Resistance pattern	MAR index	Vancomycin MIC	Vancomycin resistance genes	Biofilm-forming ability		Biofilm genes
								OD 570 ± SD*	Degree	
A1	1	Burger	11	P, ME, SAM, CXM, FEP, F, CIP, CN, DA, E, VA	0.92	64	*VanB*	0.89 ± 0.03	Strong	*icaA*, *icaB*, *icaC*, *icaD*
A2	1	Pus	10	P, ME, SAM, CXM, FEP, F, SXT, CN, E, VA	0.83	16	*VanA*, *VanB*	0.96 ± 0.04	Strong	*icaA*, *icaB*, *icaC*, *icaD*
A3	1	Sputum	10	P, ME, SAM, CXM, FEP, F, CIP, CN, E, VA	0.83	512	*VanA*, *VanB*	0.58 ± 0.02	Moderate	*icaA, icaB, icaC, icaD*
A4	1	Sputum	10	P, ME, SAM, CXM, FEP, F, CIP, CN, DA, VA	0.83	64	*VanA*	0.51 ± 0.05	Moderate	*icaA, icaD*
A5	1	Hand swab	10	P, ME, SAM, FEP, F, CIP, CN, DA, E, VA	0.83	128	*VanA*, *VanB*	0.45 ± 0.03	Moderate	*icaA, icaB, icaC, icaD*
A6	1	Shawarma	10	P, ME, SAM, CXM, FEP, CIP, CN, DA, E, VA	0.83	16	*VanA*	0.30 ± 0.01	Weak	*icaA, icaD*
A7	1	Sputum	9	P, ME, SAM, CXM, FEP, F, CN, DA, VA	0.75	32	*VanA*	0.81 ± 0.14	Strong	*icaA*, *icaB*, *icaC*, *icaD*
A8	1	Burger	9	P, ME, SAM, CXM, FEP, CIP, SXT, DA, VA	0.75	16	*VanA*	0.39 ± 0.04	Moderate	*icaA, icaB, icaC, icaD*
A9	1	Hand swab	8	P, ME, SAM, FEP, F, CIP, CN, VA	0.67	16	*VanA*	0.37 ± 0.02	Moderate	*icaB, icaC*
A10	1	Sputum	8	P, ME, SAM, CXM, FEP, F, CN, VA	0.67	256	*VanA*, *VanB*	0.92 ± 0.02	Strong	*icaA*, *icaB*, *icaC*, *icaD*,
A11	1	Burger	8	P, ME, SAM, CXM, FEP, CN, DA, E	0.67	0.5		0.42 ± 0.04	Moderate	*icaB, icaC*
A12	1	Pus	8	P, ME, SAM, CXM, FEP, F, SXT, CN	0.67	8		0.69 ± 0.06	Strong	*icaA*, *icaB*, *icaC, icaD*
A13	1	Sputum	8	P, ME, SAM, CXM, FEP, F, CIP, E	0.67	1		0.78 ± 0.1	Strong	*icaA*, *icaB*, *icaC, icaD*
A14	1	Sputum	8	P, ME, SAM, CXM, FEP, F, CIP, CN	0.67	8		0.79 ± 0.03	Strong	*icaA*, *icaB*, *icaC, icaD*
A15	1	Pus	8	P, ME, CXM, FEP, CIP, SXT, CN, E	0.67	0.5		0.48 ± 0.05	Moderate	*IcaA, icaB, icaC, icaD*
A16	1	Shawarma	8	P, ME, SAM, CXM, FEP, F, CN, E	0.67	0.25		0.41 ± 0.1	Moderate	*icaA, icaD*
A17	1	Hand swab	8	P, ME, CXM, FEP, F, CN, DA, E	0.67	0.25		0.35 ± 0.06	Moderate	*icaA, icaD*
A18	1	Pus	7	P, ME, SAM, CXM, FEP, F, SXT	0.58	4		0.32 ± 0.05	Weak	*icaD*
A19	1	Pus	7	P, ME, SAM, CXM, CIP, SXT, VA	0.58	64	*VanA*	0.47 ± 0.12	Moderate	*icaA, icaB, icaC, icaD*
A20	1	Pus	7	P, ME, SAM, CXM, FEP, CIP, VA	0.58	32	*VanA*	0.87 ± 0.03	Strong	*icaA*, *icaB*, *icaC*, *icaD*
A21	1	Pus	7	P, ME, FEP, F, CIP, CN, VA	0.58	32	*VanA*	0.44 ± 0.03	Moderate	*icaA, icaD, icaC*
A22	1	Pus	7	ME, FEP, F, CIP, SXT, DA, VA	0.58	16	*VanA*	0.43 ± 0.08	Moderate	*icaA, icaD*
A23	1	Burger	7	P, ME, SAM, FEP, CIP, CN, E	0.58	4		0.21 ± 0.06	Non-producer	
A24	1	Shawarma	7	P, ME, SAM, CXM, FEP, CIP, CN	0.58	0.5		0.38 ± 0.1	Moderate	*icaB, icaC, icaD*
A25	1	Burger	7	P, ME, SAM, CXM, FEP, F, CIP	0.58	0.25		0.40 ± 0.03	Moderate	*icaA, icaD*
A26	1	Pus	7	P, ME, SAM, CXM, FEP, F, SXT	0.58	4		0.43 ± 0.04	Moderate	*icaA, icaB, icaC*
A27	1	Sputum	7	P, ME, SAM, CXM, FEP, F, CN	0.58	8		0.45 ± 0.12	Moderate	*icaA, icaB, icaC, icaD*
A28	1	Sputum	7	P, ME, SAM, CXM, FEP, DA, E	0.58	1		0.24 ± 0.01	Weak	*icaA, icaC*
A29	1	Hand swab	7	P, ME, CXM, FEP, F, SXT, E	0.58	0.5		0.18 ± 0.04	Non-producer	
A30	1	Sputum	7	P, ME, SAM, CXM, FEP, F, VA	0.58	16	*VanA*	0.25 ± 0.03	Weak	*icaA, icaD*
A31	2	Pus	6	P, ME, SAM, CXM, FEP, CIP	0.5	0.5		0.28 ± 0.02	Weak	*icaA, icaC*
		Shawarma				0.25		0.38 ± 0.1	Moderate	*icaA, icaB, icaC, icaD*
A32	4	Pus	6	P, ME, SAM, FEP, F, CIP	0.5	1		0.77 ± 0.04	Strong	*icaA*, *icaD*
		Pus				0.5		0.25 ± 0.06	Weak	*icaA*
		sputum				1		0.46 ± 0.12	Moderate	*icaA, icaB, icaC, icaD*
		Hand swab				0.5		0.37 ± 0.02	Moderate	*icaB, icaC*
A33	2	Pus	6	P, ME, SAM, CXM, FEP, CN	0.5	1		0.42 ± 0.08	Moderate	*icaA, icaB, icaC, icaD*
		Hand swab				0.5		0.39 ± 0.01		ND
A34	1	Shawarma	6	ME, FEP, F, SXT, CN, VA	0.5	16	*VanA*	0.32 ± 0.05	Weak	*icaD*
A35	1	Shawarma	6	ME, FEP, CIP, SXT, CN, E	0.5	0.25		0.39 ± 0.2	Moderate	*icaD*
A36	1	Shawarma	6	P, ME, SAM, FEP, CIP, E	0.5	0.5		0.22 ± 0.06	Non-producer	
A 37	1	Burger	6	P, ME, SAM, FEP, F, SXT	0.5	1		0.40 ± 0.04	Moderate	*icaA, icaD*
A38	1	Hand swab	6	P, ME, FEP, F, E, VA	0.5	256	*VanA*, *VanB*	0.61 ± 0.07	Strong	*icaA*, *icaD*
A39	1	Pus	6	P, ME, SAM, FEP, CIP, CN	0.5	1		0.76 ± 0.02	Strong	*icaA*, *icaD*
A40	2	Hand swab	5	ME, FEP, F, CIP, SXT	0.42	0.5		0.39 ± 0.1	Moderate	*icaA, icaD*
						0.25		0.17 ± 0.06	Non-producer	
A41	1	Burger	5	P, ME, SAM, CXM, FEP	0.42	0.5		0.16 ± 0.01	Non-producer	
A42	1	Pus	5	P, ME, FEP, CN, E	0.42	1		0.28 ± 0.01	Weak	*icaA*
A43	1	Pus	5	ME, FEP, F, CIP, CN	0.42	0.25		0.45 ± 0.05	Moderate	ND
A44	4	Pus	4	P, ME, FEP, CIP	0.33	4		0.29 ± 0.2	Weak	ND
		Pus				4		0.41 ± 0.05	Moderate	*icaA, icaD*
		Pus				1		0.15 ± 0.04	Non-producer	
		Hand swab				0.25		0.20 ± 0.06	Non-producer	
A45	1	Pus	4	P, ME, SAM, FEP	0.33	1		0.19 ± 0.02	Non-producer	
A46	2	Hand swab	4	P, ME, FEP, DA	0.33	0.5		0.56 ± 0.04	Strong	*icaA*, *icaD*
		Pus						0.26 ± 0.06	Weak	*icaB*
A47	1	Shawarma	4	P, ME, SAM, CXM	0.33	0.25		0.21 ± 0.01	Non-producer	
A48	1	Hand swab	4	ME, CXM, FEP, DA	0.33	1		0.15 ± 0.04	Non-producer	
A49	2	Pus	3	P, ME, FEP	0.25	0.5		0.30 ± 0.1	Weak	ND
						1		0.43 ± 0.03	Moderate	*icaA, icaD*

P, penicillin; Me, methicillin; F, nitrofurantoin; SAM, ampicillin/Sulbactam; CXM, cefuroxime; CIP, ciprofloxacin; FEP, cefepime; SXT, trimethoprim/Sulfamethoxazole; CN, gentamycin; DA, clindamycin; E, erythromycin; VA, vancomycin. ND, not detected, *Average optical density value ± standard deviations (SD). Biofilm genes were assigned for biofilm-producing isolates.

### Data Analyses and Bioinformatics

The significance of association between biofilm production ability and appearance of antimicrobial resistance was determined using Fisher’s exact test applied on contingency tables. Odds ratio was estimated to give indication for the influence of biofilm production on the appearance of antimicrobial resistance. The confidence interval of odds was computed using Baptista-Pike method. Heatmap and dendrogram were generated to visualize the overall distribution and clustering of isolates based on their antimicrobial resistance profile and biofilm production ability entered as binary data (1 = present, 0 = absent). This analysis was done using “pheatmap, gplots, RColorBrewer” packages in R software (Csárdi and Nepusz, 2006). Correlation between antimicrobial resistance patterns and biofilm formation ability was determined using R packages corrplot, heatmaply, hmisc, and ggpubr (Friendly, 2002; Galili et al., 2018; Harrell, 2020) and the degree of correlation was reported following a previous study (Mukaka, 2012). To determine how isolates from human and RTE meat would be similar or dissimilar and thus estimate the potential of among-host transmission events, the antimicrobial resistance and biofilm formation profiles for each isolate were used as inputs to calculate single and averaged values of binary distance (using *dist* function within R environment) among isolates. The distances among isolates were then visualized as network diagrams, which were generated using cytoscape software version 3.8.1 (Otasek et al., 2019).

## Results

### Occurrence of *Staphylococci* in RTE Meat and Human Samples

As shown in Figure 1, we isolated 73 staphylococci isolates from the analyzed 176 samples. In the 50 isolates recovered from humans, pus samples revealed the highest number of isolates (*n* = 26) followed by hand swabs from food handlers (*n* = 14), and sputum (*n* = 10). There were 23 isolates identified from RTE meat samples, including 14 and 9 isolates from shawarma and burger, respectively. The recovered isolates, which grow onto Baird Parker and mannitol salt agar media, were Gram positive cocci forming grape-like clusters, non-spore formers, fermentative, and catalase test positive. Out of the 73 isolates, 60 (82.2%) were β-hemolytic on blood agar, grow on modified Baird Parker media, and coagulase positive, so they were identified as *S. aureus* and further confirmed by PCR amplification of *nuc* gene and MALDI TOF MS with a confidence value of 99.9%. The highest number of isolates were identified in pus (*n* = 23) followed in a descending order by hand swab (*n* = 12), sputum (*n* = 10), shawarma (*n* = 8), and burger (*n* = 7).

### Antimicrobial Resistance Profile of *S. aureus* Isolates

**FIGURE 2:**
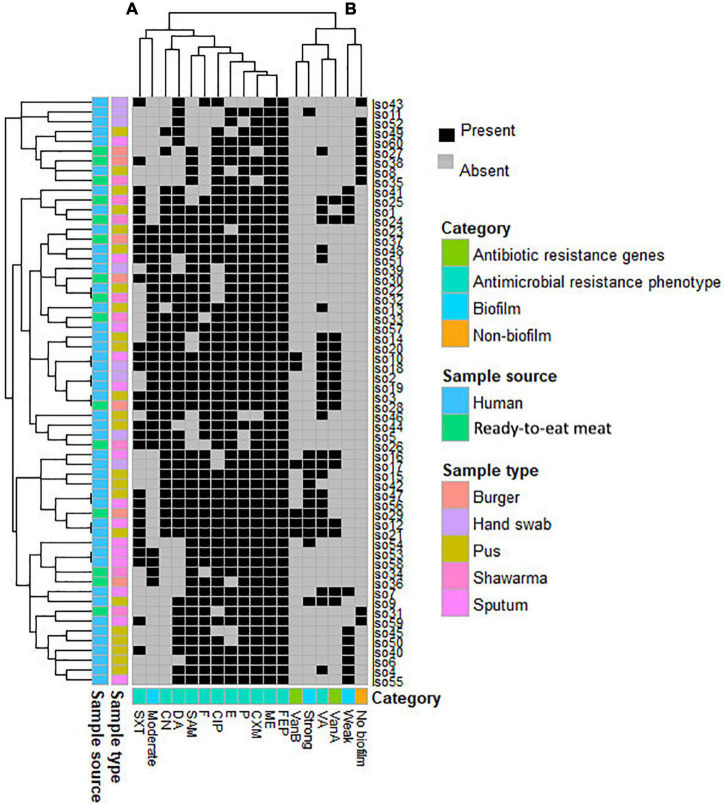
Heatmap supported by dendrogram showing the overall occurrence of antimicrobial resistance and biofilm formation profile in the study MRSA isolates (*n* = 60). Sample type and sources are shown as color-coded category on the left of the heatmap. **(A**,**B)** refer to the two clusters of the studied patterns.

The antibiogram profiles of *S. aureus* isolates revealed that all 60 isolates from RTE meat products and human samples were MDR (Table 1, Supplementary Table 2, and Figure 2), being resistant to 3–11 antimicrobials with MAR indices ranged from 0.25 to 0.92. MDR isolates showed 49 distinct antibiogram patterns. Isolates exhibiting concurrent resistance to P, ME, SAM, FEP, F, CIP (pattern A32 in Table 1) and those that showed concurrent resistance to P, ME, FEP, CIP (pattern A44 in Table 1) were the major antibiotypes being represented by 4 isolates. The isolates from RTE meat samples showed resistance to 4–11 antimicrobials with MAR indices ranged from 0.33–0.92. The hospitalized patient’s isolates (pus and sputum isolates) were resistant to 3–10 antimicrobial agents with MAR indices ranged from 0.25–0.83. Whereas the isolates from hand swabs were resistant to 4–10 antibiotics (MAR indices = 0.33–0.83). Overall, all the isolates exhibited full resistance to methicillin and harbored *mecA* gene, of which only 17 isolate (28.33%) showed resistance to vancomycin with MIC values of 16–512 μg/mL (Table 1 and Figure 1). There were 13 VRSA isolates being identified in patients’ samples (5 isolates from pus, 5 from sputum and 3 from hand swabs) and a smaller number of VRSA isolates (*n* = 4) were identified in RTE meat (shawrama [*n* = 2] and burger [*n* = 2]). As depicted in Table 1 and Supplementary Table 2, *vanA*, *vanB*, and both genes were detected in 64.7, 5.9, and 29.4% of VA-resistant isolates, respectively. The majority of MDR isolates were resistant to cefepime (96.7%) and penicillin (88.3%), and more than half (65%) were ampicillin-sulbactam resistant. The resistance to ciprofloxacin was found among 55% of isolates followed by nitrofurontoin (51.7%) and gentamicin (43.3%). The lowest resistance rates were observed for clindamycin (21.67%), sulfamethoxazole - trimethoprim (23.3%), and erythromycin (28.3%).

### Biofilm Formation Ability of MRSA and VRSA Isolates

Of the 60 MRSA isolates, 50 (83.3%) were biofilm producers and 10 (16.6%) formed no biofilm. The biofilm-producing isolates were categorized as strong (*n* = 12, 24%), moderate (*n* = 27, 54%), and weak (*n* = 11, 22%) biofilm producers (Table 1 and Supplementary Table 2). Moreover, all the 17 VRSA isolates were biofilm producers (6 strong, 8 moderate, and 3 weak). It was found that the sources of strong biofilm-producing isolates were pus (*n* = 5) and sputum (*n* = 4) from patients, hand swabs (*n* = 2) from food handlers, and burger samples (*n* = 1). The moderate biofilm producers comprised 9 from pus, 6 from hand swabs and 4 each from shawarma, burger, and sputum. Nine isolates from hospitalized patients (7 from pus and 2 from sputum) and two isolates from shawarma were weak biofilm producers (Figure 1). As depicted in Table 1 and Supplementary Tables 2, 3 the *icaADBC* biofilm-producing genes were detected in 92% of the isolates (46/50), while the *bap* gene was not found in all isolates. *icaA* was the most prevalent (76%), followed by *icaD* (74%), *icaC* (50%), and *icaB* (46%). Several biofilm gene combinations have been identified, with all *ica* genes being the most prevalent (*n* = 17), followed by *icaA* and *icaD* (*n* = 16) (Table 1).

### Diversity and Clustering Pattern of the Study Isolates

Based on the profiles of both antimicrobial resistance and biofilm formation, neither isolates from the same hosts nor isolates from the same sample source formed a single cluster (Figure 2). The analyses revealed that the 60 MRSA isolates formed 7 clusters, each cluster entails two isolates with identical profiles. Five of these clusters (71.4%) contained isolates that were all from humans and the remaining 2 clusters (28.5%) contained isolates from both humans and RTE meat. Considering all isolates, the majority of studied antimicrobial agents (*n* = 11/12, 91.6%) were positioned in a single big cluster (cluster A in Figure 2), yet only the pattern of VA resistance was separated into a different cluster (cluster B in Figure 2). The profile of moderate biofilm formation ability was also different from other biofilm profiles.

### Correlation of Antimicrobial Resistance and Biofilm Formation Ability

Irrespective of the sample type, the correlation among resistance to individual antimicrobial ranged from 0.57 to −0.010 (Supplementary Table 4). The highest positive correlation was found between resistance to SAM and P (r = 0.57, *P*-value = 1.69E-06) followed by resistance to VA and CN (r = 0.43, *P-*value = 0.0005). The occurrence of phenotypic resistance to VA was more positively correlated (r = 0.6, *P-*value = 6.07E-09) to the presence of *van A* than to that of *van B* genes (r = 0.3, *P-*value = 0.003766). The ability to form biofilm showed variable association with the appearance of antimicrobial resistance (Table 2 and Supplementary Table 4). It was found that all isolates harboring either *van A* or *van B* genes were biofilm producers and >83.3% of the isolates that showed resistance to all antimicrobials were biofilm producers. As shown in Supplementary Table 4, forming strong biofilm showed positive correlation with appearance of resistance to higher number of antimicrobials (11/12, 91.6%) than did moderate and weak biofilm formations (6/12, 50%, each). Strong biofilm was most positively correlated with the presence of *van B* gene (r = 0.38, *P-*value = 0.002). Moderate biofilm formation was correlated positively and significantly (r = 0.4, *P*-value = 0.003) with phenotypic resistance to gentamycin. Fisher’s exact test analyses revealed that the phenotypic resistance to cefuroxime, nitrofurantoin, gentamicin, and vancomycin was significantly associated with biofilm formation with variable odds ratios (Table 2). We also observed that some biofilm-producing isolates were sensitive to certain antimicrobials. For instance, 66.6% of the isolates that were phenotypically sensitive to VA and did not possess *van A* and *van B* genes (*n* = 33) were biofilm producers. Similarly, 24 (77.4%) isolates from those that were sensitive to SXT, were biofilm producers.

**TABLE 2:** The association between biofilm formation and antimicrobial resistance in the investigated 60 MRSA isolates.

Degree/antimicrobial agent		P	ME	SAM	CXM	FEP	F	CIP	SXT	CN	DA	E	VA	*MecA*	*VanA*	*VanB*
Biofilm producers	R	47*	50	41	49	50	45	42	26	35	40	46	26	50	16	6
	S	3	0	9	1	0	5	8	24	15	10	4	24	0	34	44
None-producers	R	8	10	6	7	10	3	8	3	2	6	7	1	10	1	10
	S	2	0	4	3	0	7	2	7	3	2	6	7	1	10	1
*P*-value		0.1904	>0.9999	0.2011	0.0127	>0.9999	0.0002	0.6677	0.3022	0.0047	0.2221	0.0830	0.0172	>0.9999	0.1585	0.1904
(Fisher’s exact test)*																
Odds ratio**		3.9	NA	3.0	21.0	NA	21.0	1.3	2.5	9.3	2.7	4.9	9.8	NA	4.7	3.9
Confidence		0.63–21.0	NA	0.81– 11.8	2.5– 275.5	NA	4.4– 85.6	0.24– 6.34	0.56– 9.6	1.7– 45.8	0.7246– 10.04	1.0– 22	1.2– 110	NA	0.6301– 54.02	0.6013– 21.06
Intervals																

*The data shown refer to the numbers of isolates representing each category. R = resistance, S = sensitive. P, penicillin; Me, methicillin; F, nitrofurantoin; SAM, ampicillin/Sulbactam; CXM, cefuroxime; CIP, ciprofloxacin; FEP, cefepime; SXT, trimethoprim/Sulfamethoxazole; CN, gentamycin; DA, clindamycin; E, erythromycin; VA, vancomycin. *P-value refer to the significance of association between being a biofilm- forming isolate (irrespective of degree of biofilm formation) or not and the appearance of resistance or susceptibility to the respective antibiotic **odds ratio refer to the odds of being biofilm producer when having resistance to the respective antibiotic.

### Sample Type–Dependent Association Between Antimicrobial Resistance and Biofilm Formation Ability

**FIGURE 3:**
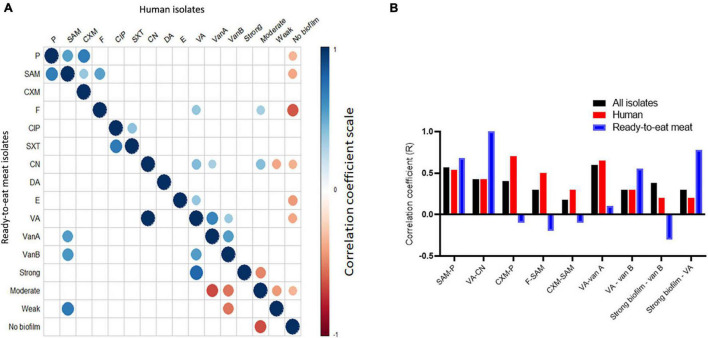
Correlation between antimicrobial resistance phenotypes and biofilm formation ability in the study isolates (*n* = 60). **(A)** Significant correlation between antimicrobial resistance and biofilm in human (upper triangle) and RTE meat (lower triangle) isolates. Blank cells refer to non-significant correlations. **(B)** Sample source-dependent variation in the correlation between antimicrobial resistance and biofilm formation.

As shown in Figures 3A,B, the correlation between antimicrobial resistance trait and biofilm formation ability was sample type-dependent (i.e., human vs. RTE meat isolates). Resistance to vancomycin and gentamycin correlated moderately positive (r = 0.4) in human isolates and strongly positive (r = 1) in RTE meat isolates. The correlation between ampicillin/sulbactam and both nitrofurantoin and cefuroxime were significantly positive in human isolates (r = 0.3 and 0.5, respectively), while this was none significant at all in RTE meat isolates. The source of the isolate has also affected the association between antimicrobial resistance and biofilm formation ability. While in RTE meat isolates moderate biofilm formation ability was significant negatively correlated with *van A* (r = −0.6) and *van B* (r = −0.5), there was no significant correlation among these pairs in human isolates. Similarly, in RTE meat isolates strong biofilm formation was significant negatively correlated with VA resistance, whereas in humans such correlation was non-significant. In human isolates, the resistance to penicillin, ampicillin/sulbactam, nitrofurantoin, gentamycin, erythromycin, and vancomycin showed variable significant negative correlation with isolates that are non-biofilm producers, whereas this correlation was non-significant in RTE meat isolates.

### Relationship Between Isolates From Various Sample Types

**FIGURE 4:**
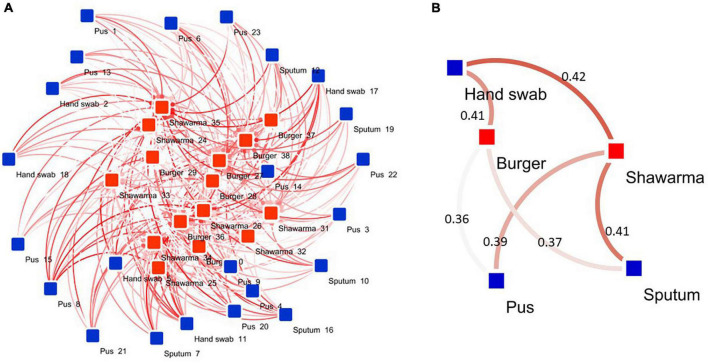
Network diagram showing the association between human and RTE meat isolates. **(A)** Binary distances between isolates (*n* = 60) belonging to various human and RTE meat sources. **(B)** Averaged binary distance between isolates from humans and RTE meat. In **(A,B)**, the binary distances were visualized as color intensity of the line between two or more isolates. The darker the color the longer the distance between isolates (longer dissimilarity). The numbers shown on the lines in **(B)** refer to average binary distance between isolates belonging to the respective pairs.

Based on averaged binary distances among isolates and network analyses, it was found that the isolates recovered from pus, sputum, and hand swabs had an average binary distance of 0.36, 0.37, and 0.41, respectively, to those from burger. Furthermore, isolates from pus, sputum, and hand swabs had an average binary distance of 0.39, 0.41, and 0.42, respectively, to the isolates from shawarma (Figure 4).

## Discussion

Nowadays, there is an increased trend of RTE food afforded by infinite number of restaurants and street vendors all over the world, notably in Egypt. While this provides the advantage of fast food, it comes with the challenge and risk of bacterial infection especially these RTE products are not further exposed to heating. Raw meat contaminated with *S. aureus* is considered to be a major cause of food poisoning worldwide (de Boer et al., 2009; Wang et al., 2014; Raji et al., 2016). The risk of infection is being increased when the contaminated meat is not cooked sufficiently or RTE food is contaminated with this bacterium by cross-contamination (Wang et al., 2017). Recently, RTE food products have been reported as reservoirs for methicillin-resistant coagulase-negative staphylococci (MRCONS) (Yang et al., 2017), but the occurrence, resistance profile, and biofilm formation ability of methicillin-resistant coagulase-positive staphylococci (MRCOPS) and VRSA have received much less attention and require further in-depth investigation. Indeed, the available studies in Egypt have provided detailed characterization of MRSA strains in raw beef meat (Osman et al., 2015), and the available studies on RTE meat have described the occurrence of antimicrobial resistance without analyzing the biofilm formation ability, although being an important survival feature and none of these studies have analyzed the clustering and association of isolates coming from humans and RTE meat (Saad et al., 2019; Mahros et al., 2021). Here we tried to fill in these gaps by characterizing a set of MRCOPS and VRSA isolates from human and RTE meat with respect to their occurrence, antibiotic resistance profile, and biofilm formation ability.

In this study, 23.4% of RTE meat samples tested positive for *S. aureus*. These findings are higher than that documented in other studies on RTE meat products associated with foodborne illness where isolation rates of *S. aureus* were 9.5% in southern Taiwan (Wei et al., 2006), 11.8% in China (Yang et al., 2016), and 15.4% in Taiwan (Fang et al., 2003), but lower than the rates reported in chicken (40.9%) and pork meat (34.4%) from Shaanxi Province, China (Wang et al., 2014), and Qalubiya Governorate, Egypt (50.8%) (Saad et al., 2019). Our findings also revealed non-significant difference (*P* > 0.05) in the occurrence of *S. aureus* in shawarma (36.8%) and burger sandwiches (34.6%), which were lower than that reported in burger (43.3%) and nearly similar to that observed in shawarma (36.6%) in another Egyptian study (Saad et al., 2019). The differences in the microbiological quality of raw ingredient, the utensils and equipment used for food preparation, cooking, the presence of seasoning ingredients, isolation methods may contribute to the observed differences in the bacterial detection rate (Daelman et al., 2013; Wang et al., 2014). Our study reported a higher frequency of MRSA isolates from the examined COPS isolates recovered from shawarma and burger sandwiches (100%) than that obtained previously in beef burger and hot dog sandwiches (22.2%) (Mahros et al., 2021) and in other RTE meat products (25%) (Saad et al., 2019). This suggests the increased occurrence of MRSA in this kind of RTE meat. The current study showed that high number of MRSA strains were also VRSA (17/60, 28.3%). This is already higher than that obtained previously in Egypt by Mahros et al. (2021). The prevalence of MRSA (23.4%, 15 isolates/64 sample) and VRSA (6.25%, 4/64) in RTE meat samples in our study was higher than that observed (6.4% MRSA and 0% VRSA) in RTE meat (cooked pork, chicken, and duck) from China (Yang et al., 2016). In addition, a lower prevalence of MRSA (2.3% in chicken meat and 0.6% in pork meat) and VRSA (0%) were detected by Wang et al. (2014). However, Al-Amery et al. (2019) found that 4% (8/200) of camel meat samples from Egypt were positive for VRSA.

Food can become contaminated with *S. aureus* or MRSA from the food-producing animals or from infected food handlers during various processing stages (de Boer et al., 2009; Crago et al., 2012; Al-Amery et al., 2019). Since *S. aureus* is vulnerable to destruction by heating and sanitizing agents, its presence in RTE foods might be related to human contamination rather that contamination from animal origin and is indicative of poor sanitation during processing (U.S. Food and Drug Administration, 1998; Fontes et al., 2013). Generally, infected food handlers are reported to be responsible for causing 20% of bacterial foodborne illnesses. Here, the carriage rate of *S. aureus* in food handlers was 30% (12 isolates/40 samples), all isolates were MRSA and 25% of which was VRSA, indicating that food handlers, which are clinically healthy could pose a serious risk to public health and food safety. Other researches from different countries reported various rates of MRSA carriage on hands and noses of food handlers ranging from 1.6% in Bosnia and Herzegovina (Uzunoviæ et al., 2013), 28.6% in Brazil (Ferreira et al., 2014), and to 79% in Turkey (Sezer et al., 2015). Moreover, Ferreira et al. (2014) reported that 72.9 and 82.5% of the isolates from the handlers’ hand and nose were VRSA, respectively. However, VRSA isolates were not identified in the study done in Bosnia and Herzegovina (Uzunoviæ et al., 2013). This variation could be based on different factors including health conditions of food handlers, cleaning habits, level of education, the state of development of the country, and the regulations on inquest (Sezer et al., 2015). This suggests the importance of the continuous survey and proper hygiene of the food handlers, which could decrease contamination by this pathogen and minimize the risk of disease.

Because MRSA and VRSA have been identified in RTE food worldwide, it is supposed that it could be transmitted to humans, causing illness. This study showed that 45.8% of patients’ samples were positive for *S. aureus*, all isolates were of MRSA type and 30.3% of patients’ isolates were VRSA. In support of our findings, a prospective surveillance study in Egypt has reported an upsurge in the prevalence of HA-MRSA and VRSA as the rate of MRSA has doubled from 48.6 to 86.8% in a 8-year period (from 2005 to 2013) and the proportion of VRSA has increased from 4.2 to 25.8% during the same period (Abdel-Maksoud et al., 2016). Conversely, comparatively lower proportions have been reported in other countries: *S. aureus* isolation rate was 14.3%, MRSA was 17.5%, and VRSA was 5.1% in Ethiopia (Dilnessa and Bitew, 2016) and 63.1, 72, and 15.9%, respectively, in Eritrea (Garoy et al., 2019). The observed variation is possibly attributed to the study population and duration, types of the specimen, and the used laboratory and control procedures.

MRSA isolates pose more risk if they resist treatment by antimicrobials. Alarmingly, all MRSA isolates characterized in the current study from all sources were MDR showing resistance to clinically important antimicrobials including cefepime, penicillin, and ampicillin-sulbactam, ciprofloxacin, nitrofurontoin, gentamicin, vancomycin, erythromycin, sulfamethoxazole - trimethoprim, and clindamycin. Other studies done in Egypt also reported higher resistance rates of HA-MRSA to clindamycin (56%), gentamicin (80.7%), and erythromycin (64.4%) (Abdel-Maksoud et al., 2016). Nearly similar results were observed for penicillin and vancomycin among MRSA isolates from Ethiopian patients but higher resistance was noted for erythromycin and trimethoprim-sulfamethoxazole (Dilnessa and Bitew, 2016). Other authors have reported considerably lower frequency of vancomycin and gentamicin resistance in Eritrea (Garoy et al., 2019).

The higher resistance rates of MRSA isolates cultured from RTE meat for cefuroxime, ciprofloxacin, gentamicin, and erythromycin than those isolated from humans is consonance with results from previous studies in retail foods (Wang et al., 2014) and RTE foods (Yang et al., 2016) in China and food animals in Tanzania (Mdegela et al., 2021). Our findings are even more serious in terms of public health hazard because shawarma and burger sandwiches are consumed without further cooking, which if happened would eliminate or reduce the load of pathogen. Overall, the high frequency of MDR isolates in the study area has been related to prevail, excessive use and imprudent use of antibiotics, including in livestock husbandry, self-medication, and substandard infection control and prevention practices (Garoy et al., 2019). Accordingly, governments and individuals should pay attention to prevent further spread of MRSA and VRSA.

In addition to exhibiting antimicrobial resistance, MRSA isolates are able to produce biofilm as a defense mechanism enabling further survival in an infection niche (Archer et al., 2011). It is plausible to assume that a successful isolate would be able to form biofilm and resist antimicrobials. Our study supports this idea, where MRSA isolates with strong biofilm producers were resistant to 91.6% of the studied antimicrobials. There was also significant association between phenotypic resistance to cefuroxime, nitrofurantoin, gentamicin, vancomycin and biofilm formation. In agreement, Weigel and coauthors reported that vancomycin resistance is associated with and enhanced by the microenvironment of a biofilm (Weigel et al., 2007). These observations are similar to those reported in a study in Italy, in which food isolates of MRSA (milk and pork) have the ability to produce biofilms, as has been found previously for clinical MRSA (Vergara et al., 2017).

In *S. aureus*, the *ica* genes play a key role in biofilm formation. Strains with the *icaADBC* cluster especially *icaA* and *icaD* have previously been identified as potential biofilm producers (Arciola et al., 2001). These genes were detected in 92% of the biofilm-producing isolates with *icaA* and *icaD* being the most prevalent (76 and 74%, respectively). Our findings are consistent with the results of prior studies that found the *ica* genes in all isolates (Chen et al., 2020) especially both *icaA* and *icaD* genes (Szweda et al., 2012; Dai et al., 2019). However, Bissong and Ateba (2020) detected the *ica* genes in 75.3% of isolates with a lower proportion of *icaA* and *icaD* combination. These findings imply that *S. aureus* strains may differ in their ability to produce biofilms depending on their source (human or animal) and geographical origin. The results of this study, like those of previous research, indicated that the *bap* gene was not detected in biofilm-producing isolates (Szweda et al., 2012; Dai et al., 2019; Chen et al., 2020). Although not all genes involved in biofilm formation were examined, this might imply that the *ica*-dependent pathway is predominantly responsible for adhesion and biofilm formation in these isolates. Other biofilm-associated genes may be responsible for biofilm formation in isolates that tested negative for any of the genes investigated.

Based on the biofilm-forming capability in chronic *S. aureus*-related infection, some chemoprophylaxis procedures such as vaccine candidates were recently documented (Mirzaei et al., 2016, 2019, 2021; Bahonar et al., 2019; Gholami et al., 2019). Gholami et al. (2019) declared that a vaccine containing a mixture of PIA and glycerol teichoic acid (Gly-TA), biofilm related macromolecules, could be effective in preventing *S. aureus* biofilm formation and protect against infection. Furthermore, PIA-rSesC conjugate vaccine has been developed to raise antibodies against PIA in order to eliminate biofilm-forming *S. aureus* and *S. epidermidis* (Bahonar et al., 2019; Mirzaei et al., 2019).

In the absence of advanced sequencing technologies, a comprehensive and deep characterization of bacteria, and thus similarity among isolates from various hosts, is difficult to attain. However, studying targeted antimicrobial resistance and biofilm formation ability in a set of isolates could unravel important isolate’s characteristics, especially looking at homogenicity among isolates within and among hosts as shown in our previous investigations in *Campylobacter jejuni* (Abd El-Hamid et al., 2019), *Aeromonas hydrophila* (Tartor et al., 2021), and *Escherichia coli* (Ramadan et al., 2020). This could initiate hypothesizing some zoonotic aspects and transmission scenarios across hosts. Here we showed that considering both antimicrobial resistance and biofilm formation potential, all isolates within most of the *S. aureus* clusters (i.e., the 5 clusters) were from human and none of the RTE meat isolates have identical profiles and thus formed no clusters. In addition, only two clusters were formed that contain mixed RTE meat and human isolates. In support of this, our correlation analyses revealed certain degree of disparity between human and RTE meat isolates, where gene correlation differed between both isolates in the two sources (Figure 3). While this should be interpreted using more isolates, this initially reflects the differential use of antimicrobials in each source (i.e., humans vs. RTE meat) (Pokharel et al., 2020). It also suggests the uniqueness of within-hosts isolates and that isolates within a single host (e.g., humans in the current study) could have developed similar fitness strategies such as antimicrobial resistance or biofilm formation ability. There are some clues from previous comprehensive genomic analyses that the majority of the animal-associated *S. aureus* clustered into animal-specific lineages a part from human lineages (Sung et al., 2008). Moreover, human lineages of *S. aureus* were rarely found in animals (Sakwinska et al., 2011). This was also supported by our observation of the host-dependent correlation between presence of antimicrobial resistance and biofilm formation ability (Figure 3). These observations motivated us to perform further analyses to determine the extent of similarities and dissimilarities among isolates from human and RTE meat using estimates of average binary distance among all isolates as done previously (Abd El-Hamid et al., 2019). Our observation of high binary distances between human isolates and either burger or shawarma isolates supports the uniqueness of within-host bacterial population.

A shortcoming of this study is the small number of examined samples, which was reflected on the small number of obtained isolates ready for characterization and analyses. However, it was convenient at least as an initial study on these two important sources, to balance the sample number, the multi-sample type nature included in the study and the needed analyses. We acknowledge that the traditional characterization methods applied here are not by any way considered as alternative for advance genomics tools (e.g., whole genome sequencing), and it is becoming clear that introducing modern genomics is vital and inevitable if it comes to comprehensive characterization of bacterial population, which we are planning for in future investigation.

## Conclusion

The high prevalence of MRSA and VRSA isolates in shawarma and burger sandwiches in the present study suggests that RTE meat could be a potential source of MRSA and VRSA isolates with significant clinical relevance. The ability of MRSA and VRSA isolates to produce biofilms and the presence of high rates of antimicrobial resistance amongst the isolates highlight the danger posed by these potentially virulent microorganisms persisting in RTE meat, food handlers, and patients. Taken together, good hygiene practices and antimicrobial surveillance plans should be strictly implemented along the food chain to reduce the risk of colonization and dissemination of MRSA and VRSA biofilm-producing strains.

## Data Availability Statement

The original contributions presented in the study are included in the article/Supplementary Material, further inquiries can be directed to the corresponding author/s.

## Author Contributions

RE-M, EA, SE-S, GE, AA, AM, and SA performed investigation, practical work, and supervision. MS performed the statistical and bioinformatics analyses, prepared the figures, and participated with YT in formulating the tables. MS and YT investigated the data and designed the research manuscript. MS, with inputs from YT, took the lead in directing the research manuscript. YT wrote the initial draft of the manuscript. All authors revised the manuscript and agreed to the final version.

## Conflict of Interest

The authors declare that the research was conducted in the absence of any commercial or financial relationships that could be construed as a potential conflict of interest.

## Publisher’s Note

All claims expressed in this article are solely those of the authors and do not necessarily represent those of their affiliated organizations, or those of the publisher, the editors and the reviewers. Any product that may be evaluated in this article, or claim that may be made by its manufacturer, is not guaranteed or endorsed by the publisher.

## References

[B1] Abd El-Hamid M. I. Abd El-Aziz N. K. Samir M. El-Naenaeey E. Y. Abo Remela E. M. Mosbah R. A. (2019). Genetic diversity of *Campylobacter jejuni* isolated from avian and human sources in Egypt. *Front. Microbiol.* 10:2353. 10.3389/fmicb.2019.02353 31681217 PMC6813243

[B2] Abdel-Maksoud M. El-Shokry M. Ismail G. Hafez S. El-Kholy A. Attia E. (2016). Methicillin-resistant *Staphylococcus aureus* recovered from healthcare- and community-associated infections in Egypt. *Int. J. Bacteriol.* 2016 1–5. 10.1155/2016/5751785 27433480 PMC4940577

[B3] Al-Amery K. Elhariri M. Elsayed A. El-Moghazy G. Elhelw R. El-Mahallawy H. (2019). Vancomycin-resistant *Staphylococcus aureus* isolated from camel meat and slaughterhouse workers in Egypt. *Antimicrob. Resist. Infect. Control* 8:129. 10.1186/s13756-019-0585-4 31404199 PMC6683426

[B4] Amberpet R. Sistla S. Sugumar M. Nagasundaram N. Manoharan M. Parija S. (2019). Detection of heterogeneous vancomycin-intermediate *Staphylococcus aureus*: a preliminary report from south India. *Indian J. Med. Res.* 150 194–198. 10.4103/ijmr.IJMR_1976_17 31670275 PMC6829776

[B5] Anjum M. F. Marco-Jimenez F. Duncan D. Marín C. Smith R. P. Evans S. J. (2019). Livestock-associated methicillin-resistant *Staphylococcus aureus* from animals and animal products in the UK. *Front. Microbiol.* 10:2136. 10.3389/fmicb.2019.02136 31572341 PMC6751287

[B6] Archer N. K. Mazaitis M. J. William Costerton J. Leid J. G. Powers M. E. Shirtliff M. E. (2011). *Staphylococcus aureus* biofilms: properties, regulation and roles in human disease. *Virulence* 2 445–459. 10.4161/viru.2.5.17724 21921685 PMC3322633

[B7] Arciola C. R. Baldassarri L. Montanaro L. (2001). Presence of *icaA* and *icaD* genes and slime production in a collection of staphylococcal strains from catheter-associated infections. *J. Clin. Microbiol.* 39 2151–2156. 11376050 10.1128/JCM.39.6.2151-2156.2001 PMC88104

[B8] Aureus G. (2016). *National Standard of the People’s Republic of China National Food Safety Standard Food Microbiological Examination: Staphylococcus Aureus.* Rome: FAO.

[B9] Avila-Novoa M. G. González-Gómez J.-P. Guerrero-Medina P. J. Cardona-López M. A. Ibarra-Velazquez L. M. Velazquez-Suarez N. Y. (2021). *Staphylococcus aureus* and methicillin-resistant *Staphylococcus aureus* (MRSA) strains isolated from dairy products: relationship of *ica*-dependent/independent and components of biofilms produced in vitro. *Int. Dairy J.* 119:105066. 10.1016/j.idairyj.2021.105066

[B10] Bahonar S. Ghazvinian M. Haghshenas M. R. Goli H. R. Mirzaei B. (2019). Purification of PIA and rSesC as putative vaccine candidates against *Staphylococcus aureus*. *Rep. Biochem. Mol. Biol.* 8 161–167. 31832440 PMC6844615

[B11] Bissong M. E. A. Ateba C. N. (2020). Genotypic and phenotypic evaluation of biofilm production and antimicrobial resistance in *Staphylococcus aureus* isolated from milk, north west province, South Africa. *Antibiotics* 9:156. 10.3390/antibiotics9040156 32252278 PMC7235893

[B12] Brakstad O. G. Aasbakk K. Maeland J. A. (1992). Detection of *Staphylococcus aureus* by polymerase chain reaction amplification of the *nuc* gene. *J. Clin. Microbiol.* 30 1654–1660. 10.1128/jcm.30.7.1654-1660.1992 1629319 PMC265359

[B13] Chajecka-Wierzchowska W. Zadernowska A. Nalepa B. Sierpińska M. Laniewska-Trokenheim L. (2015). Coagulase-negative staphylococci (CoNS) isolated from ready-to-eat food of animal origin - Phenotypic and genotypic antibiotic resistance. *Food Microbiol.* 46 222–226. 10.1016/j.fm.2014.08.001 25475289

[B14] Cheesbrough M. (2006). *District Laboratory Practice in Tropical Countries*, Second Edn. Cambridge, MA: Cambridge University Press.

[B15] Chen C. J. Huang Y. C. (2014). New epidemiology of *Staphylococcus aureus* infection in Asia. *Clin. Microbiol. Infect.* 20 605–623. 10.1111/1469-0691.12705 24888414

[B16] Chen Q. Xie S. Lou X. Cheng S. Liu X. Zheng W. (2020). Biofilm formation and prevalence of adhesion genes among *Staphylococcus aureus* isolates from different food sources. *MicrobiolOpen* 9:e946. 10.1002/mbo3.946 PMC6957440 31769202

[B17] CLSI (2018). *Performance Standards for Antimicrobial Susceptibility Testing*, 28th Edn. Wayne: Clinical and Laboratory Standards Institute.

[B18] Craft K. M. Nguyen J. M. Berg L. J. Townsend S. D. (2019). Methicillin-resistant: *Staphylococcus aureus* (MRSA): antibiotic-resistance and the biofilm phenotype. *Med. Chem. Commun.* 10 1231–1241. 10.1039/c9md00044e 31534648 PMC6748282

[B19] Crago B. Ferrato C. Drews S. J. Svenson L. W. Tyrrell G. Louie M. (2012). Prevalence of *Staphylococcus aureus* and methicillin-resistant *S. aureus* (MRSA) in food samples associated with foodborne illness in Alberta, Canada from 2007 to 2010. *Food Microbiol.* 32 202–205. 10.1016/j.fm.2012.04.012 22850394

[B20] Csárdi G. Nepusz T. (2006). The igraph software package for complex network research. *InterJournal* 1695 1–9.

[B21] Cucarella C. Tormo M. A. Ubeda C. Trotonda M. P. Monzón M. Peris C. (2004). Role of biofilm—Associated protein bap in the pathogenesis of bovine *Staphylococcus aureus*. *Infect. Immun.* 72 2177–2185. 15039341 10.1128/IAI.72.4.2177-2185.2004 PMC375157

[B22] Daelman J. Jacxsens L. Lahou E. Devlieghere F. Uyttendaele M. (2013). Assessment of the microbial safety and quality of cooked chilled foods and their production process. *Int. J. Food Microbiol.* 160 193–200. 10.1016/j.ijfoodmicro.2012.10.010 23290224

[B23] Dai J. Wu S. Huang J. Wu Q. Zhang F. Zhang J. (2019). Prevalence and characterization of *Staphylococcus aureus* isolated from pasteurized milk in China. *Front. Microbiol.* 10:641. 10.3389/fmicb.2019.00641 31001225 PMC6454862

[B24] de Boer E. Zwartkruis-Nahuis J. T. M. Wit B. Huijsdens X. W. de Neeling A. J. Bosch T. (2009). Prevalence of methicillin-resistant *Staphylococcus aureus* in meat. *Int. J. Food Microbiol.* 134 52–56. 10.1016/j.ijfoodmicro.2008.12.007 19144432

[B25] Dilnessa T. Bitew A. (2016). Prevalence and antimicrobial susceptibility pattern of methicillin resistant *Staphylococcus aureus* isolated from clinical samples at Yekatit 12 Hospital Medical College, Addis Ababa, Ethiopia. *BMC Infect. Dis.* 16:398. 10.1186/s12879-016-1742-5 27506613 PMC4977752

[B26] Fang T. J. Wei Q. K. Liao C. W. Hung M. J. Wang T. H. (2003). Microbiological quality of 18^°^C ready-to-eat food products sold in Taiwan. *Int. J. Food Microbiol.* 80 241–250. 10.1016/S0168-1605(02)00172-1 12423926

[B27] Ferreira J. S. Costa W. L. R. Cerqueira E. S. Carvalho J. S. Oliveira L. C. Almeida R. C. C. (2014). Food handler-associated methicillin-resistant *Staphylococcus aureus* in public hospitals in Salvador, Brazil. *Food Control* 37 395–400. 10.1016/j.foodcont.2013.09.062

[B28] Fontes C. O. Silva V. L. de Paiva M. R. B. Garcia R. A. Resende J. A. Ferreira-Machado A. B. (2013). Prevalence, antimicrobial resistance, and virulence characteristics of *mecA*-encoding coagulase-negative Staphylococci isolated from soft cheese in Brazil. *J. Food Sci.* 78 M594–M599. 10.1111/1750-3841.12088 23488927

[B29] Friendly M. (2002). Corrgrams. *Am. Stat.* 56 316–324. 10.1198/000313002533 12611515

[B30] Galili T. O’Callaghan A. Sidi J. Sievert C. (2018). Heatmaply: an R package for creating interactive cluster heatmaps for online publishing. *Bioinformatics* 34 1600–1602. 10.1093/bioinformatics/btx657 29069305 PMC5925766

[B31] Garoy E. Y. Gebreab Y. B. Achila O. O. Tekeste D. G. Kesete R. Ghirmay R. (2019). Methicillin-resistant *Staphylococcus aureus* (MRSA): prevalence and antimicrobial sensitivity pattern among patients - A multicenter study in Asmara, Eritrea. *Can. J. Infect. Dis. Med. Microbiol.* 2019:8321834. 10.1155/2019/8321834 30881532 PMC6381584

[B32] Gharieb R. M. A. Saad M. F. Mohamed A. S. Tartor Y. H. (2020). Characterization of two novel lytic bacteriophages for reducing biofilms of zoonotic multidrug-resistant *Staphylococcus aureus* and controlling their growth in milk. *LWT Food Sci. Technol.* 124:109145. 10.1016/j.lwt.2020.109145

[B33] Gholami S. A. Goli H. R. Haghshenas M. R. Mirzaei B. (2019). Evaluation of polysaccharide intercellular adhesion (PIA) and glycerol teichoic acid (Gly-TA) arisen antibodies to prevention of biofilm formation in *Staphylococcus aureus* and *Staphylococcus epidermidis* strains. *BMC Res. Notes* 12:691. 10.1186/s13104-019-4736-8 31653277 PMC6815028

[B34] Guo Y. Song G. Sun M. Wang J. Wang Y. (2020). Prevalence and therapies of antibiotic-resistance in *Staphylococcus aureus*. *Front. Cell. Infect. Microbiol.* 10:107. 10.3389/fcimb.2020.00107 32257966 PMC7089872

[B35] Hadjirin N. F. Lay E. M. Paterson G. K. Harrison E. M. Peacock S. J. Parkhill J. (2015). Detection of livestock-associated meticillin-resistant *Staphylococcus aureus* CC398 in retail pork, United Kingdom, february 2015. *Eurosurveillance* 20 1–4. 10.2807/1560-7917.es2015.20.24.21156 26111237 PMC4841384

[B36] Harrell F. E. Jr. (2020). *Package “Hmisc” Version 4.6-0.*

[B37] Hiramatsu K. Katayama Y. Matsuo M. Sasaki T. Morimoto Y. Sekiguchi A. (2014). Multi-drug-resistant *Staphylococcus aureus* and future chemotherapy. *J. Infect. Chemother.* 20 593–601. 10.1016/j.jiac.2014.08.001 25172776

[B38] Holmes N. E. Tong S. Y. C. Davis J. S. Hal S. J. V. (2015). Treatment of methicillin-resistant *Staphylococcus aureus*: vancomycin and beyond. *Semin. Respir. Crit. Care Med.* 36 17–30. 10.1055/s-0034-1397040 25643268

[B39] Kariyama R. Mitsuhata R. Chow J. W. Clewell D. B. Kumon H. (2000). Simple and reliable multiplex PCR assay for surveillance isolates of vancomycin-resistant enterococci. *J. Clin. Microbiol.* 38 3092–3095. 10.1128/jcm.38.8.3092-3095.2000 10921985 PMC87194

[B40] Kiem S. Oh W. S. Peck K. R. Lee N. Y. Lee J. Y. Song J. H. (2004). Phase variation of biofilm formation in *Staphylococcus aureus* by IS 256 insertion and its impact on the capacity adhering to polyurethane surface. *J. Korean Med. Sci.* 19 779–782. 10.3346/jkms.2004.19.6.779 15608385 PMC2816298

[B41] Krumperman P. (1983). Multiple antibiotic resistance indexing of *Escherichia coli* to identify high-risk sources of fecal contamination of foods. *Appl. Environ. Microbiol.* 46 165–170. 10.1007/s11356-014-3887-3 6351743 PMC239283

[B42] Li H. Andersen P. S. Stegger M. Sieber R. N. Ingmer H. Staubrand N. (2019). Antimicrobial resistance and virulence gene profiles of Methicillin-Resistant and -susceptible *Staphylococcus aureus* from food products in Denmark. *Front. Microbiol.* 10:2681. 10.3389/fmicb.2019.02681 31920996 PMC6920630

[B43] Luo Y. Siu G. K. H. Yeung A. S. F. Chen J. H. K. Ho P. L. Leung K. W. (2015). Performance of the VITEK MS matrix-assisted laser desorption ionization-time of flight mass spectrometry system for rapid bacterial identification in two diagnostic centres in China. *J. Med. Microbiol.* 64 18–24. 10.1099/jmm.0.080317-0 25418737

[B44] Mahros M. A. Abd-Elghany S. M. Sallam K. I. (2021). Multidrug-, methicillin-, and vancomycin-resistant *Staphylococcus aureus* isolated from ready-to-eat meat sandwiches: an ongoing food and public health concern. *Int. J. Food Microbiol.* 346:109165. 10.1016/j.ijfoodmicro.2021.109165 33770679

[B45] McClure J. A. Conly J. M. Lau V. Elsayed S. Louie T. Hutchins W. (2006). Novel multiplex PCR assay for detection of the staphylococcal virulence marker Panton-Valentine leukocidin genes and simultaneous discrimination of methicillin-susceptible from -resistant staphylococci. *J. Clin. Microbiol.* 44 1141–1144. 10.1128/JCM.44.3.1141-1144.2006 16517915 PMC1393128

[B46] McKenney D. Ubner J. H. Muller E. Wang Y. Goldmann D. A. Pier G. B. (1998). The *ica* locus of *Staphylococcus epidermidis* encodes production of the capsular polysaccharide/adhesion. *Infect. Immun.* 66 4711–4720. 10.1128/IAI.66.10.4711-4720.1998 9746568 PMC108579

[B47] Mdegela R. H. Mwakapeje E. R. Rubegwa B. Gebeyehu D. T. Niyigena S. Msambichaka V. (2021). Antimicrobial use, residues, resistance and governance in the food and agriculture sectors, Tanzania. *Antibiotics* 10 1–23. 10.3390/antibiotics10040454 33923689 PMC8073917

[B48] Mirzaei B. Babaei R. Valinejad S. (2021). Staphylococcal vaccine antigens related to biofilm formation. *Hum. Vaccin. Immunother.* 17 293–303. 10.1080/21645515.2020.1767449 32498595 PMC7872035

[B49] Mirzaei B. Moosavi S. F. Babaei R. Siadat S. D. Vaziri F. Shahrooei M. (2016). Purification and evaluation of polysaccharide intercellular adhesion (PIA) antigen from *Staphylococcus epidermidis*. *Curr. Microbiol.* 73 611–617. 10.1007/s00284-016-1098-5 27460584

[B50] Mirzaei B. Mousavi S. F. Babaei R. Bahonar S. Siadat S. D. Shafiee Ardestani M. (2019). Synthesis of conjugated PIA-rSesC and immunological evaluation against biofilm-forming *Staphylococcus epidermidis*. *J. Med. Microbiol.* 68 791–802. 10.1099/jmm.0.000910 30990402

[B51] Mossong J. Decruyenaere F. Moris G. Ragimbeau C. Olinger C. M. Johler S. (2015). Investigation of a staphylococcal food poisoning outbreak combining case–control, traditional typing and whole genome sequencing methods, Luxembourg, June 2014. *Eurosurveillance* 20:30059. 10.2807/1560-7917.ES.2015.20.45.30059 26608881

[B52] Mukaka M. M. (2012). Statistics corner: a guide to appropriate use of correlation coefficient in medical research. *Malawi Med. J.* 24 69–71. 23638278 PMC3576830

[B53] Osman K. M. Amer A. M. Badr J. M. Saad A. S. A. (2015). Prevalence and antimicrobial resistance profile of *Staphylococcus* species in chicken and beef raw meat in Egypt. *Foodborne Pathog. Dis.* 12 406–413. 10.1089/fpd.2014.1882 25789407

[B54] Otasek D. Morris J. H. Bouças J. Pico A. R. Demchak B. (2019). Cytoscape automation: empowering workflow-based network analysis. *Genome Biol.* 20 1–15. 10.1186/s13059-019-1758-4 31477170 PMC6717989

[B55] Otto M. (2014). *Staphylococcus aureus* toxins. *Curr. Opin. Microbiol.* 17 32–37. 10.1016/j.mib.2013.11.004 24581690 PMC3942668

[B56] Patel R. Uhl J. R. Kohner P. Hopkins M. K. Cockerill F. R. (1997). Multiplex PCR detection of *vanA, vanB, vanC-1*, and *vanC-2/3* genes in *Enterococci*. *J. Clin. Microbiol.* 35 703–707. 10.1128/jcm.35.3.703-707.1997 9041416 PMC229654

[B57] Periasamy S. Joo H.-S. Duong A. C. Bach T.-H. L. Tan V. Y. Chatterjee S. S. (2012). How *Staphylococcus aureus* biofilms develop their characteristic structure. *PNAS* 109 1281–1286. 10.1073/pnas.1115006109 22232686 PMC3268330

[B58] Pokharel S. Shrestha P. Adhikari B. (2020). Antimicrobial use in food animals and human health: time to implement “One Health” approach. *Antimicrob. Resist. Infect. Control* 9 1–5. 10.1186/s13756-020-00847-x 33160396 PMC7648983

[B59] Raji M. A. Garaween G. Ehricht R. Monecke S. Shibl A. M. Senok A. (2016). Genetic characterization of *Staphylococcus aureus* isolated from retail meat in Riyadh, Saudi Arabia. *Front. Microbiol.* 7:911. 10.3389/fmicb.2016.00911 27375611 PMC4899468

[B60] Ramadan H. Jackson C. R. Frye J. G. Hiott L. M. Samir M. Awad A. (2020). Antimicrobial resistance, genetic diversity and multilocus sequence typing of *Escherichia coli* from humans, retail chicken and ground beef in Egypt. *Pathogens* 9:357. 10.3390/pathogens9050357 32397188 PMC7281645

[B61] Roberson J. R. Fox L. K. Hancock D. D. Besser T. E. (1992). Evaluation of methods for differentiation of coagulase-positive staphylococci. *J. Clin. Microbiol.* 30 3217–3219. 1452705 10.1128/jcm.30.12.3217-3219.1992 PMC270634

[B62] Saad M. S. Hassanin F. S. Shaltout F. A. Nassif M. Z. Seif M. Z. (2019). Prevalence of methicillin-resistant *Staphylococcus aureus* in some ready-to-eat meat products. *Am. J. Biomed. Sci. Res.* 4 461–465. 10.34297/ajbsr.2019.04.000855

[B63] Sakwinska O. Giddey M. Moreillon M. Morisset D. Waldvogel A. Moreillon P. (2011). *Staphylococcus aureus* host range and human-bovine host shift. *Appl. Environ. Microbiol.* 77 5908–5915. 10.1128/AEM.00238-11 21742927 PMC3165375

[B64] Sezer Ç Özgür Ç Aksem A. Leyla V. (2015). Food handlers: a bridge in the journey of enterotoxigenic MRSA in food. *J. Verbraucherschutz Leb.* 10 123–129. 10.1007/s00003-015-0939-7

[B65] Stepanovic S. Vukovic D. Hola V. Di Bonaventura G. Djukic S. Cirkovic I. (2007). Quantification of biofilm in microtiter plates: overview of testing conditions and practical recommendations for assessment of biofilm production by staphylococci. *APMIS* 115 891–899. 10.1111/j.1600-0463.2007.apm_630.x 17696944

[B66] Sung J. M. L. Lloyd D. H. Lindsay J. A. (2008). *Staphylococcus aureus* host specificity: comparative genomics of human versus animal isolates by multi-strain microarray. *Microbiology* 154 1949–1959. 10.1099/mic.0.2007/015289-0 18599823

[B67] Szweda P. Schielmann M. Milewski S. Frankowska A. Jakubczak A. (2012). Biofilm production and presence of *ica* and *bap* genes in *Staphylococcus aureus* strains isolated from cows with mastitis in the eastern Poland. *Pol. J. Microbiol.* 61 65–69. 22708349

[B68] Tartor Y. H. El-Naenaeey E.-S. Y. Abdallah H. M. Samir M. Yassen M. M. Abdelwahab A. M. (2021). Virulotyping and genetic diversity of *Aeromonas hydrophila* isolated from Nile tilapia (*Oreochromis niloticus*) in aquaculture farms in Egypt. *Aquaculture* 541:736781. 10.1016/j.aquaculture.2021.736781

[B69] U.S. Food and Drug Administration (1998). *Bacteriological Analytical Manual*, eighth Edn. Arlington, VA: Association of Official Analytical Chemists.

[B70] Uzunoviæ S. Ibrahimagiæ A. Kamberoviæ F. Rijnders M. I. A. Stobberingh E. E. (2013). Molecular characterization of methicillin-susceptible and methicillin-resistant *Staphylococcus aureus* in food handlers in Bosnia and Herzegovina. *Open Infect. Dis. J.* 7 15–20. 10.2174/1874279301307010015

[B71] Vasudevan P. Nair M. K. Annamalai T. Venkitanarayanan K. S. (2003). Phenotypic and genotypic characterization of bovine mastitis isolates of *Staphylococcus aureus* for biofilm formation. *Vet. Microbiol.* 92 179–185. 10.1016/s0378-1135(02)00360-7 12488081

[B72] Vergara A. Normanno G. Di Ciccio P. Pedonese F. Nuvoloni R. Parisi A. (2017). Biofilm formation and its relationship with the molecular characteristics of food-related Methicillin-Resistant *Staphylococcus aureus* (MRSA). *J. Food Sci.* 82 2364–2370. 10.1111/1750-3841.13846 28892140

[B73] Wang W. Baloch Z. Jiang T. Zhang C. Peng Z. Li F. (2017). Enterotoxigenicity and antimicrobial resistance of *Staphylococcus aureus* isolated from retail food in China. *Front. Microbiol.* 8:2256. 10.3389/fmicb.2017.02256 29209290 PMC5702451

[B74] Wang X. Li G. Xia X. Yang B. Xi M. Meng J. (2014). Antimicrobial susceptibility and molecular typing of methicillin-resistant *Staphylococcus aureus* in retail foods in Shaanxi, China. *Foodborne Pathog. Dis.* 11 281–286. 10.1089/fpd.2013.1643 24404781

[B75] Weber J. T. (2005). Community-associated methicillin-resistant *Staphylococcus aureus*. *Clin. Infect. Dis.* 41 S269–S272. 10.1086/430788 16032563

[B76] Wei Q. K. Hwang S. L. Chen T. R. (2006). Microbiological quality of ready-to-eat food products in southern Taiwan. *J. Food Drug Anal.* 14 68–73. 10.38212/2224-6614.2512

[B77] Weigel L. M. Donlan R. M. Shin D. H. Jensen B. Clark N. C. McDougal L. K. (2007). High-level vancomycin-resistant *Staphylococcus aureus* isolates associated with a polymicrobial biofilm. *Antimicrob. Agents Chemother.* 51 231–238. 10.1128/AAC.00576-06 17074796 PMC1797660

[B78] Yang T.-Y. Hung W.-W. Lin L. Hung W.-C. Tseng S.-P. (2017). *mecA*-related structure in methicillin-resistant coagulase-negative staphylococci from street food in Taiwan. *Sci. Rep.* 9:42205. 10.1038/srep42205 28181543 PMC5299846

[B79] Yang X. Zhang J. Yu S. Wu Q. Guo W. Huang J. (2016). Prevalence of *Staphylococcus aureus* and methicillin-resistant *Staphylococcus aureus* in retail ready-to-eat foods in China. *Front. Microbiol.* 7:816. 10.3389/fmicb.2016.00816 27375562 PMC4895929

