# Supplementary material for: Methicillin- and Vancomycin-Resistant Staphylococcus aureus From Humans and Ready-To-Eat Meat: Characterization of Antimicrobial Resistance and Biofilm Formation Ability
Source: Front Microbiol. 2022 Feb 8;12:735494. doi: 10.3389/fmicb.2021.735494 (PMC8861318; doi:10.3389/fmicb.2021.735494)
Supplement: Supplementary file 1 [file Table_1.doc]

**Supplementary Table 1.** Sequences of oligonucleotide primers used in the study

| **Target gene** | **Primer sequences (5’-3’)** | **Product sizes (bp)** | **References** |
| --- | --- | --- | --- |
| *nuc* | F: GCGATTGATGGTGATACGGTT | 270 | Brakstad et al. (1992) |
|  | R: AGCCAAGCCTTGACGAACTAAAGC |  |  |
| *mecA* | F: GTAGAAATGACTGAACGTCCGATAA | 310 | McClure et al. (2006) |
|  | R: CCAATTCCACATTGTTTCGGTCTAA |  |  |
| *vanA* | F: CATGACGTATCGGTAAAATC | 885 | Patel et al. (1997) |
|  | R: ACCGGGCAGRGTATTGAC |  |  |
| *vanB* | F: GTGACAAACCGGAGGCGAGGA | 433 | Kariyama *et al*. (2000) |
|  | R: CCGCCATCCTCCTGCAAAAAA |  |  |
|  | R: AGTGCCTTCCCAAACCTTTT |  |  |
| *icaA* | F: CCTAACTAACGAAAGGTAG | 1315 | Vasudevan, et al. (2003) |
|  | R: AAGATATAGCGATAAGTG C |  |  |
| *icaD* | F: AAACGTAAGAGAGGTGG | 381 |  |
|  | R: GGCAATATGATCAAGATA |  |  |
| *icaB* | F: AGAATCGTGAAGTATAGAAAATT | 880 | Kiem et al. (2004) |
|  | R: TCTAATCTTTTTCATGGAATCCGT |  |  |
| *icaC* | F: ATGGGACGGATTCCATGAAAAAGA | 1,066 |  |
|  | R: TAATAAGCATTAATGTTCAATT |  |  |
| *bap* | F: CCCTATATCGAAGGTGTAGAATTGCAC | 971 | Cucarella et al. (2001) |
|  | R: GCTGTTGAAGTTAATACTGTACCTGC |  |  |
