# Supplementary material for: Methicillin- and Vancomycin-Resistant Staphylococcus aureus From Humans and Ready-To-Eat Meat: Characterization of Antimicrobial Resistance and Biofilm Formation Ability
Source: Front Microbiol. 2022 Feb 8;12:735494. doi: 10.3389/fmicb.2021.735494 (PMC8861318; doi:10.3389/fmicb.2021.735494)
Supplement: Supplementary file 3 [file Table_3.doc]

**Supplementary table S3.** Occurrence of biofilm-associated genes in MRSA isolates with various biofilm formation ability.

| **Biofilm formation** | **Number (%) of isolates harboring biofilm genes** | | | | |
| --- | --- | --- | --- | --- | --- |
|  | ***icaA*** | ***icaB*** | ***icaC*** | ***icaD*** | ***bap*** |
| Strong (n = 12) | 12 (100) | 8 (66.7) | 8 (66.7) | 12 (100) | 0 |
| Moderate (n = 27) | 20 (74.1) | 14 (51.9) | 15 (55.6) | 21 (77.8) | 0 |
| Weak (n = 11) | 6 (54.5) | 1 (9.1) | 2 (18.2) | 4 (36.4) | 0 |
| Total (n = 50) | 38 (76) | 23 (46) | 25 (50) | 37 (74) | 0 |
